# The Awareness and Knowledge of Retinopathy of Prematurity Among Pediatricians in Saudi Arabia

**DOI:** 10.7759/cureus.60754

**Published:** 2024-05-21

**Authors:** Bahaaeldin H Abdelhalim, Abdulmalik Alotaibi, Naif Alruwais, Faisal Alotaibi, Abdulaziz Alotaibi, Abdulaziz Alosaimi, Fahad Alsahli

**Affiliations:** 1 Ophthalmology, Shaqra University, Shaqra, SAU

**Keywords:** tractional retinal detachment, fundus screening, retinopathy of prematurity, neonatal care, pre-term

## Abstract

Background: Retinopathy of prematurity (ROP) affects premature low birth-weight infants with potentially blinding complications. Early diagnosis and treatment for indicated cases are essential to prevent unfavorable effects of the disease.

Objectives: To determine the awareness and the level of knowledge of ROP among pediatricians in all regions of Saudi Arabia who worked at neonatal intensive care units.

Design and setting: This was a cross-sectional study. This study was undertaken in the Kingdom of Saudi Arabia.

Materials and methods: A semi-self-structured online questionnaire was designed to study the awareness and knowledge of ROP. The questionnaire contained items related to demographic data, participants’ level of understanding and knowledge of ROP, and items related to hospital facilities, the availability of a trained ophthalmologist, and the source of knowledge.

Main outcome measures: The awareness and knowledge of ROP among pediatricians of the Kingdom of Saudi Arabia.

Sample size: The study included 145 pediatricians.

Results: Awareness of ROP was found in 138 participants (95.2%). Seven participants (4.8%) were not aware of ROP and were excluded from further analysis. Of the participants, 116 (84.0%), 127 (92.0%), and 130 (94.3%) had knowledge about the stages, treatment, and precautions of treatment of ROP, respectively. Of the participants, 77 (55.8%), 63 (45.7%), 113 (81.9%), and 56 (40.6%) gave the correct answer about the indications of fundus examination, exact time of the first fundus examination, place of fundus examination, and minimum number of screening fundus examination for ROP, respectively.

Conclusions: The awareness and knowledge of ROP among pediatricians of Saudi Arabia is good, but knowledge about the indications and proper time of first referral to an ophthalmologist should be improved.

## Introduction

Retinopathy of prematurity (ROP) is a fibrovascular proliferation of the peripheral nonvascularized retina of preterm infants that may lead to total tractional retinal detachment and may be a cause of irreversible bilateral blindness if not diagnosed and treated properly. For this reason, preterm infants have to be screened and followed up following a proper protocol to diagnose a clinically significant disease and refer them for treatment if indicated. The established risk factors for ROP are prematurity, low birth weight, a complex hospital course, and prolonged supplemental oxygen [1-3]. At least two screening fundus examinations should be done for all infants with a birth weight of ≤1500 g or a gestational age of 30 weeks or less, and selected infants with a birth weight between 1500 and 2000 g or a gestational age of >30 weeks who are believed by their attending pediatrician or neonatologist to be at risk for ROP [1-4]. Screening examination should be done by a trained ophthalmologist in a neonatal unit for in-patient babies but discharged babies can be examined in a neonatal or ophthalmic unit [1,5]. ROP runs through five stages. Stage 1 is a demarcation line between vascularized and nonvascularized retina; stage 2, a ridge that has height and width, occupies a volume; stage 3, when the ridge has extraretinal fibrovascular proliferation; stages 4 and 5, subtotal and total retinal detachment [1,2]. Plus disease indicates a more florid form. It occurs with increasing retinal vascular dilation and tortuosity, engorgement of iris vessels, pupillary rigidity, and vitreous haze.

This study aimed to determine the level of awareness and knowledge of ROP among pediatricians of Saudi Arabia who worked in neonatal intensive care units (NICU).

## Materials and methods

This cross-sectional study was conducted in Saudi Arabia from February to May 2023 and included 145 pediatricians who worked at Saudi Arabian hospitals and had experience in the NICU. All pediatric levels from residents to consultants were included. The responses were collected from all Saudi major regions (Central, Western, Eastern, Southern, and Northern areas). Data were collected using an anonymous, semi-self-structured online questionnaire that was developed and modified after an extensive literature review and expert consultation and then translated into Arabic. The questionnaire was divided into three parts: the first part contained items related to demographic data and one item related to awareness of ROP; the second part consisted of nine sections that measured participants’ level of understanding and knowledge of ROP; and the third part contained three items related to hospital facilities, the availability of a trained ophthalmologist, and the source of knowledge. The validity of the questionnaire was assessed by five experts from the ophthalmology and pediatric departments for meaning and content sufficiency. It was modified based on the expert suggestions. Reliability was assessed using a pilot sample of 30 participants to determine whether there were any unclear questions. These participants were excluded from the main study. The reliability index was 0.85. The questionnaire was shared using social media platforms (e.g., WhatsApp and Twitter) and by data collectors. The inclusion criteria were any pediatrician working in Saudi Arabian hospitals who had at least one year of experience in the NICU. Any participant who did not complete the questionnaire or did not provide consent for participation was excluded. The participants were included consecutively if they met the inclusion criteria.

Ethical considerations

Participation was completely voluntary, and participants could respond at their available time and convenience. Each participant provided informed consent. Confidentiality was maintained strictly to ensure data privacy and protect participants’ information. The participants had the right to withdraw from the study at any time. The study was approved by the Scientific Research Ethics Committee at Shaqra University. The study adhered to the guidelines of the Declaration of Helsinki.

Statistical analysis

After data collection, the data were extracted, revised, coded, and fed to statistical software SPSS version 22 (IBM Corp., Armonk, NY). Data were expressed as numbers and percentages and the chi-square test was used to compare groups. All statistical analysis was done using two-tailed tests. A p-value less than 0.05 was considered statistically significant.

## Results

The study included 145 participants, including 83 males (57.2%) and 62 females (42.8%). A total of 138 participants (95.2%) were aware of ROP and seven (4.8%) were not aware of ROP. The participants who had no knowledge (five specialists and two residents) were excluded from further analysis. Of the participants, 30 (21.74%) got their knowledge about ROP from undergraduate study, 89 (64.49%) from postgraduate study, and 19 (13.77%) from self-study. The demographic data of participants are summarized in Table 1. Of the participants, 37.0% were from the Central region of Saudi Arabia, 20.3% from the Southern region, 19.6% from the Western region, 15.2% from the Eastern region, and 8.0% from the Northern region.

**Table 1:** Demographic data of Saudi pediatricians (n = 138) in the survey for retinopathy of prematurity.

Parameters	No. (%)
Sex	
Male	80 (57.97)
Female	58 (42.03)
Age, years	
25-29	50 (36.23)
30-34	33 (23.91)
35-39	20 (14.49)
>40	35 (25.36)
Experience in NICU, years	
<1	21 (15.22)
1-4	72 (52.17)
5-9	17 (12.32)
>10	28 (20.29)
Practice level	
Residents	52 (37.68)
Specialists	42 (30.43)
Consultants	44 (31.88)
Residency	
Central region	51 (36.96)
Eastern region	21 (15.22)
Northern region	11 (7.97)
Southern region	28 (20.29)
Western region	27 (19.27)
Workplace	
Governmental hospital	127 (92.03)
Private hospital	11 (7.79)

Regarding the knowledge of ROP, 116 (84.0%), 127 (92.0%), and 130 (94.3%) participants had knowledge about the stages, treatment, and precautions of treatment of ROP, respectively (Table 2). Regarding the awareness of ROP, 77 (55.8%), 63 (45.7%), 113 (81.9%), and 56 (40.6%) participants gave the correct answer about the indications of fundus examination, exact time of the first fundus examination, place of fundus examination, and minimum number of screening fundus examination for ROP, respectively. A total of 110 (79.71%) participants answered that their hospital had a trained ophthalmologist for the examination of ROP, while 28 (20.29%) participants reported that their hospital had no trained ophthalmologists.

**Table 2:** Knowledge and awareness of Saudi pediatricians (n = 138) about retinopathy of prematurity.

Parameters	No. (%)
Indications of fundus examination	
Correct answer	77 (55.80)
Incorrect answer	51 (36.96)
No idea	10 (7.25)
Timing of the first fundus examination	
Correct answer	63 (45.65)
Incorrect answer	64 (46.38
No idea	11 (7.79)
Place of fundus examination	
Correct answer	113 (81.88)
Incorrect answer	19 (13.77)
No idea	6 (4.35)
Minimum number of screening fundus examination	
Correct answer	56 (40.58)
Incorrect answer	49 (35.50)
No idea	33 (23.91)
Are there any precautions for fundus examination?	
Yes	130 (94.30)
No	8 (5.80)
Knowledge about the stages	
Yes	120 (86.96)
No	18 (13.04)
Knowledge about the treatment	
Yes	127 (92.03)
No	11 (7.79)

Table 3 shows the comparison between groups with knowledge of ROP. The significant findings indicated that individuals aged 30-34 years possessed a significantly higher level of knowledge regarding the timing of the first fundus examination. Similarly, individuals aged 35-39 years exhibited a significantly higher level of knowledge of the timing of the first fundus examination compared to other age groups. Pediatricians with less than one year of experience in the NICU had a significantly lower level of knowledge about the timing of the first fundus examination than other age groups. Residents had significantly higher levels of knowledge about the least number of screening fundus examinations and specialists had significantly lower levels of knowledge about the least number of screening fundus examinations. Pediatricians of the Eastern region of Saudi Arabia had significantly higher levels of knowledge about the timing of the first fundus examination and the least number of screening fundus examinations. Pediatricians of the Southern region had a significantly lower level of knowledge about the least number of screening fundus examinations and pediatricians of the Western Region had a significantly lower level of knowledge about the least number of screening fundus examinations. No significant changes between groups in other parameters were found.

**Table 3:** Comparison between groups about knowledge of retinopathy of prematurity (ROP). * = ꭓ^2^.

Parameters	Indications of screening fundus examination		Timing of first fundus examination	
	No. (%)	P*	No. (%)	P*
Sex				
Male, n = 80	45 (56.25)		38 (47.50)	
Female, n = 58	32 (55.17)	0.90	25 (43.10)	0.61
Age, years				
25-29, n = 50	26 (52.00)	0.50	18 (36.00)	0.09
30-34, n = 33	19 (57.58)	0.81	21 (63.64)	0.02
35-39, n = 20	8 (40.00)	0.12	5 (20.00)	0.045
>40, n = 35	24 (68.57)	0.08	19 (54.29)	0.24
Experience in NICU, years				
<1, n = 21	13 (61.90)	0.54	4 (19.05)	<0.01
1-4, n = 72	36 (50.00)	0.15	37 (51.39)	0.1
5-9, n = 17	8 (47.06)	0.44	6 (35.29)	0.36
>10, n = 28	20 (71.43)	0.06	16 (57.14)	0.17
Level				
Residents, n = 52	25 (48.08)	0.16	26 (50.00)	0.43
Specialists, n = 42	24 (57.14)	0.83	18 (42.86)	0.66
Consultants, n = 44	28 (63.64)	0.20	19 (43.18)	0.69
Residency				
Central region, n =51	31 (60.78)	0.37	19 (37.25)	0.13
Eastern region, n = 21	9 (42.86)	0.19	16 (76.19)	<0.01
Northern region, n = 11	5 (45.45)	0.47	7 (63.64)	0.21
Southern region, n = 28	13 (46.43)	0.26	14 (50.00)	0.60
Western region, n = 27	19 (70.37)	0.09	7 (25.93)	0.02
Caring a baby with ROP				
Yes, n = 111	64 (57.66)		53 (47.75)	
No, n = 27	13 (48.15)	0.37	10 (37.04)	0.32
The hospital has a protocol for ROP				
Yes, n = 125	69 (55.20)		61 (48.80)	
No, n = 13	8 (61.54)	0.66	2 (15.38)	0.02

Table 4 shows the comparison between groups about the knowledge of stages and treatment of ROP. Pediatricians in the Northern region had significantly higher knowledge about the treatment of ROP. Pediatricians who did not care for a baby in the NICU had significantly lower knowledge about the treatment and stages of ROP. No significant changes between groups in other parameters were found.

**Table 4:** Comparison between groups about awareness of stages and treatment of retinopathy of prematurity (ROP). * = ꭓ^2^.

Parameters	Awareness of stages of ROP		Awareness of the treatment of ROP	
	No. (%)	P*	No. (%)	P-value*
Sex				
Male, n = 80	72 (90.00)		76 (95.00)	
Female, n = 58	48 (82.76)	0.16	51 (87.93)	0.13
Age, years				
25-29, n = 50	43 (86.00)	0.80	47 (94.00)	0.52
30-34, n = 33	28 (84.85)	0.68	28 (84.85)	0.08
35-39, n = 20	16 (80.00)	0.32	18 (90.00)	0.72
>40, n = 35	33 (94.29)	0.14	34 (97.14)	0.20
Experience in NICU, years				
<1, n = 21	18 (85.71)	0.85	18 (85.71)	0.25
1-4, n = 72	61 (84.72)	0.42	68 (94.44)	0.27
5-9, n = 17	14 (82.35)	0.55	14 (8.35)	0.12
>10, n = 28	27 (96.43)	0.14	27 (96.43)	0.34
Practice level				
Residents, n = 52	44 (84.62)	0.53	47 (90.38)	0.58
Specialists, n = 42	37 (88.10)	0.79	39 (92.86)	0.81
Consultants, n = 44	39 (88.64)	0.69	41 (93.18)	0.73
Residency				
Central region, n =51	47 (92.16)	0.16	49 (96.08)	0.18
Eastern region, n = 21	18 (85.71)	0.85	21 (100)	0.14
Northern region, n = 11	8 (72.73)	0.14	8 (72.73)	0.01
Southern region, n = 28	24 (85.71)	0.83	24 (85.71)	0.18
Western region, n = 27	23 (85.19)	0.76	25 (92.59)	0.90
Caring a baby with ROP				
Yes, n = 111	106 (95.50)		107 (96.40)	
No, n = 27	14 (51.85)	<0.01	20 (74.04)	<0.01
The hospital has a protocol for ROP				
Yes, n = 125	109 (87.20)		116 (92.80)	
No, n = 13	11 (84.62)	0.71	11 (84.62)	0.30

## Discussion

This cross-sectional study was undertaken to determine the awareness and knowledge among pediatricians of ROP in Saudi Arabia. ROP may lead to unfavorable outcomes if not diagnosed and properly managed with a medicolegal implication on the organization and included physicians. More than 95% of our participants were aware of ROP, which is a very favorable result. Comparable results were obtained in one study from South India [6]. Contrary to these results, another study in South India found the awareness of pediatricians about ROP is only 65% [7]. The most important tasks of neonatologists regarding ROP are the knowledge of the indications of screening and the appropriate time of referral of an infant with ROP to the ophthalmologist. Further management will be undertaken by the ophthalmologist. The precise correct answer was obtained in 77% for the indications, and 63% for the appropriate time of referral of ROP. These results should be increased to include most (if not all) neonatologists. Current available screening protocols in the United States and the United Kingdom, although effective and sensitive in identifying a clinically significant disease, may not be generalizable to other areas with different neonatal care protocols [8]. Different regions with different standards of neonatal care may need to evolve their own protocol for screening ROP.

In this study, we found pediatricians with less than one year of experience in the NICU have significantly lower knowledge levels regarding the timing of the first fundus examination. These pediatricians should be under the supervision of more experienced ones. The pediatricians working in hospitals that have a protocol for screening ROP have significantly higher knowledge levels about ROP screening than others who have no protocol. Hospitals caring for neonates should have teaching programs to teach newcomers more about ROP and should have a protocol for screening ROP in order not to miss a case for referral at a proper time. The pediatricians working in the Western region significantly have a lower knowledge level regarding the time of the first referral of a case of ROP. This area may need special care for increasing the knowledge of ROP. Regarding the indications of screening of ROP, there were no significant differences in the knowledge between all studied groups.

The pediatricians who are involved in caring for a baby have a higher knowledge level about the stages and treatment of ROP may be because of their contact and discussion with ophthalmologists.

A trained ophthalmologist should always be available all the time for ROP examination. Some hospitals in this study do not have a trained ophthalmologist, which may lead to a delay in the diagnosis of clinically significant disease. With increasing neonatal care and the living rate of premature infants, ROP is a worldwide problem. Previous studies have reported a 33-73% incidence of ROP (at any stage) in preterm infants less than 27-28 weeks and severe ROP in 16-35% [9-13]. Awareness and knowledge of ROP may be given to all healthcare providers, including nurses [14,15]. All hospitals that provide neonatal care should have a protocol for screening and management of ROP and a protocol for training new neonatologists. Also, these hospitals should have a trained ophthalmologist and well-equipped neonatal units for fundus examination of premature babies. Teaching ROP may be important for all healthcare providers. This may be through undergraduate courses in ophthalmology and pediatrics, postgraduate study, and/or self-study.

Anyhow, this study has some limitations. The results were limited to Saudi Arabia, so they cannot be generalized. Only a few responses were provided from the Northern region. The level of hospitals, whether primary, secondary, or tertiary, was not evaluated. Pediatricians, whether Saudi or non-Saudi, were not evaluated.

## Conclusions

Pediatricians in Saudi Arabia demonstrate a high-level understanding of ROP; however, there is room for enhancement in their comprehension regarding the appropriate indications and timing for the initial referral to an ophthalmologist. All neonatologists need to have sufficient knowledge about the screening of ROP and the exact time of the first referral of a baby with ROP to the ophthalmologist. This will lead to proper management and prevention of the unfavorable sequel of the disease and also avoid unnecessary examination that can cause significant morbidity in neonates. Also, hospitals should work to decrease risk factors of ROP hoping to prevent or decrease the development of ROP.

## Appendices

Questionnaire for awareness and knowledge of retinopathy of prematurity

A. Demographic data

Sex

Male

Female

Age, years

25-29

30-34

35-39

>40

Years of experience in NICU

<1

1-4

5-9

>10

Do you have knowledge about ROP?

Yes

No

Practice level

Residents

Specialists

Consultants

Residency

Central region

Eastern region

Northern region

Southern region

Western region

Workplace

Governmental hospital

Private hospital

Have you taken care of a baby with ROP?

Yes

No

B. The level of understanding and knowledge of ROP

What are the main indications of fundus examination in preterm infants?

Frequent RBC transfusion

Supplemental O2

Birth weight <1500 g and gestational age ≤30 weeks

No idea

When should the first fundus examination be performed?

Depends on gestational age

4-6 weeks of chronologic age

6-12 weeks of chronologic age

No idea

Where should the fundus examination of a preterm infant be done?

Neonatal unit

Ophthalmology unit

Family medicine unit

No idea

What is the minimum number of screening fundus examinations for ROP?

2

3

4

No idea

Are there any precautions during the fundus examination of a preterm infant?

Yes

No

Do you have any idea about the stages of ROP and the threshold disease?

Yes

No

Do you have any idea about the treatment of ROP?

Yes

No

C. Hospital facilities and source of knowledge

Does your hospital have any facilities for the treatment of ROP?

Yes

No

In your hospital, is there a trained ophthalmologist for screening ROP?

Yes

No

From where did you get the knowledge about ROP?

Undergraduate study

Postgraduate study

Self-study
